# Characterizing isomiR variants within the microRNA‐34/449 family

**DOI:** 10.1002/1873-3468.12595

**Published:** 2017-02-28

**Authors:** Olivier Mercey, Alexandra Popa, Amélie Cavard, Agnès Paquet, Benoît Chevalier, Nicolas Pons, Virginie Magnone, Joséphine Zangari, Patrick Brest, Laure‐Emmanuelle Zaragosi, Gilles Ponzio, Kevin Lebrigand, Pascal Barbry, Brice Marcet

**Affiliations:** ^1^ CNRS, IPMCUniversité Côte d'AzurSophia‐AntipolisValbonneFrance; ^2^ CNRS, INSERM, IRCAN, FHU‐OncoAgeUniversité Côte d'AzurSophia‐AntipolisValbonneFrance

**Keywords:** isomiR, microRNA, miR‐34, miR‐449, miRBase, multiciliated cell

## Abstract

miR‐34/449 microRNAs are conserved regulators of multiciliated cell differentiation. Here, we evidence and characterize expression of two isomiR variant sequences from the miR‐34/449 family in human airway epithelial cells. These isomiRs differ from their canonical counterparts miR‐34b and miR‐449c by one supplemental uridine at their 5′‐end, leading to a one‐base shift in their seed region. Overexpression of canonical miR‐34/449 or 5′‐isomiR‐34/449 induces distinct gene expression profiles and biological effects. However, some target transcripts and functional activities are shared by both canonical microRNAs and isomiRs. Indeed, both repress important targets that result in cell cycle blockage and Notch pathway inhibition. Our findings suggest that 5′‐isomiR‐34/449 may represent additional mechanisms by which miR‐34/449 family finely controls several pathways to drive multiciliogenesis.

## Abbreviations

**AGO**, Argonaute protein

**ALI**, air–liquid interface

**F‐actin**, filamentous actin

**FUCCI**, fluorescence ubiquitin cell cycle indicator

**HAECs**, human airway epithelial cells

**miR‐449**, miR‐449a, miR‐449b, and miR‐449c

**miRNAs or miR**, microRNAs

**RISC**, RNA‐induced silencing complex

MicroRNAs (miRNAs or miRs) are endogenous, small noncoding RNAs 1, 2, which act as major post‐transcriptional regulators of gene expression in multicellular organisms 3, and are involved in specific pathophysiological events 4, 5, 6. Mature miRNAs are short 20–24 nucleotides single‐stranded RNAs. A short stretch of 6–8 nucleotides at the 5′‐end, termed the ‘seed region’ 7, 8, plays a major role in the interaction with target messenger RNAs (mRNAs), usually in their 3′‐untranslated region (3′‐UTR). miRNAs regulate the expression of their target transcripts through mechanisms involving control of mRNA stability or translational efficiency 1. The miRNA/mRNA interaction is driven by complex rules that go far beyond perfect complementarities, making sometimes *in silico* identification of targets inaccurate. Regulation by miRNAs represents a powerful mechanism to accurately fine‐tune gene expression of several *a priori* distinct biological processes. The growing interest for miRNAs has led to the creation of a specialized database, miRBase 9, which current release (version 21) gathers information on 35 828 mature miRNA products across 223 species. This database has been instrumental to disseminate useful information about miRNAs.

miRNA genomic clusters are often cotranscribed as related miRNAs 10, 11 which share the same seed region and/or belong to functionally related miRNA families 12, 13. A typical example is given by the miR‐34/449 superfamily which exhibits extensive genomic redundancy by encompassing six homologous miRNAs (miR‐34a, miR‐34b/c, and miR‐449a/b/c) located on three distinct genomic *loci* 14, 15, 16, 17, 18, 19. miR‐34b/c and miR‐449a/b/c are abundantly expressed in multiciliated cells in which they act as pleiotropic regulators of vertebrate multiciliogenesis, through their action at several cellular levels: arrest of the cell cycle and inhibition of the Notch pathway 15, maturation and docking of basal bodies 17, as well as induction of the apical actin meshwork formation 19. Mice deficient in either miR‐34a, miR‐34b/c, and miR‐449a/b/c or in miR‐34b/c and miR‐449a/b/c exhibit motile cilia impairment together with respiratory and fertility defects, while mice invalidated only for one of these miRNAs exhibit no discernible phenotype 14, 16, 17, 18, 20. This functional redundancy is likely due to strong sequence homologies between miR‐34/449 miRNAs, which extend further than the seed region.

miRNA sequences may undergo several modifications such as uridylation, adenylation, RNA editing, or methylation that affect their properties and functions 21. Recent advances in high throughput sequencing technologies have also refined the definition of miRNA variants, named 5′‐ or 3′‐isomiRs, which differ from the canonical miRNAs by one or more bases at the 3′‐ and/or 5′‐end. Seldom, the sequence reported in miRBase accounts for a very small fraction of a given miRNA 22, 23, 24, 25, 26, 27, 28, 29, 30. Although isomiRs have been reported in many datasets, their biological relevance and contribution to the functional repertoire of a specific miRNA gene remain controversial 29, 31, 32, 33, 34. Noisy measurements can hardly account for the high frequency of some isomiRs which are not randomly distributed 35, suggesting these isomiRs have a regulated production and a biological relevance.

The present study started as we pointed out that hsa‐miR‐34b‐5p (miR‐34b) and hsa‐miR‐449c‐5p (miR‐449c) have distinct seed region according to the sequences reported in miRBase. They differ from the other members of the family (hsa‐miR‐34a, ‐34c‐5p, ‐449a, and ‐449b) by the addition of one uridine (‘U’) at the 5′‐end of the two sequences. Using small RNA sequencing of human airway epithelial cell (HAEC) primary cultures, we show here that miRNA sequences referenced in miRBase for miR‐34b or miR‐449c actually represent minor variants that we propose to consider as isomiRs (5′‐isomiR‐34b and 5′‐isomiR‐449c). On the opposite, the most abundantly expressed sequences related to miR‐34b and miR‐449c share exactly the same seed region with other canonical members of the miR‐34/449 family. Overall, our findings provide evidence that canonical miR‐34/449 and 5′‐isomiR‐34/449 exhibit dramatic changes in the mRNA target recognition and can also display some functional differences/complementarities, as both block cell cycle and Notch pathway, whereas they differently affect actin network reorganization.

## Materials and methods

### Tissue samples and primary culture of human airway epithelial cells

Inferior nasal turbinates were collected from patients who underwent surgical intervention for nasal obstruction or septoplasty (provided by Pr Castillo, Nice University Hospital, Nice, France). The use of human tissues was authorized by the bioethical law 94‐654 of the French Public Health Code after written consent from the patients. Primary HAEC cultures were performed as previously described 15, 19.

### Ectopic expression of miRNAs and isomiRs

Primary HAEC, A549, or HEK293T cells were grown to 30% confluency on plastic plates or glass coverslip. Hsa‐miR‐34b‐5p (5′‐AGGCAGUGUCAUUAGCUGAUUGU‐3′) and hsa‐miR‐449c‐5p (5′‐AGGCAGUGUAUUGCUAGCGGCUGU‐3′) custom mimics were designed from the predominant form in small RNA sequencing data (Ambion, Life Technologies, Carlsbad, CA, USA). Cells were then transfected with synthetic negative control miRNA (miR‐Neg, Ambion, Life Technologies) or synthetic miRNAs (miRBase references for hsa‐miR‐34a, ‐34c‐5p, ‐449a, and ‐449b, and newly synthesized sequences for hsa‐miR‐34b‐5p and hsa‐miR‐449c‐5p, Ambion, Life Technologies) and synthetic isomiRs (5′‐isomiR‐34b and 5′‐isomiR‐449c, referenced as miR‐34b and miR‐449c in miRBase, Ambion, Life Technologies) at 10 nm using Lipofectamine RNAi Max Reagent (Invitrogen, Carlsbad, CA, USA) in OPTIMEM (Invitrogen) according to the manufacturer's instructions. Total RNAs and proteins were extracted after 48 h and 72 h after transfection, respectively.

### RNA/small RNA sequencing

Total RNAs were isolated from primary undifferentiated, fully differentiated, miR‐, and 5′‐isomiR‐transfected HAEC cultures using QIAcube and miRNeasy kit from QIAGEN according to the manufacturer's instructions. Total RNAs were then quantified using NanoDrop 1000 Spectrophotometer (Thermo Fisher Scientific, Waltham, MA, USA), and integrity of samples (RIN > 8) was evaluated using RNA nanochips on the Agilent 2100 Bioanalyzer Instrument (Agilent Technologies, Santa Clara, CA, USA). For RNA sequencing, polyA RNAs were purified using dynabeads mRNA purification kit (Invitrogen) and tagmented for 9 min at 95 °C. To test molecular cloning bias, synthetic mature miRNAs or isomiRs (10 pg) were mixed with 1.5 μg of a complex RNA mixture of 3T3 cells just before performing small RNA sequencing. RNA and small RNA libraries were generated using the NEB next small library prep set for SOLiD (New England Biolabs, Ipswich, MA, USA) and sequenced on the Applied Biosystems SOLiD 5500 wildfire system following the manufacturer's instructions. All data generated from RNA sequencing were stored on microarray and next generation sequencing information system Mediante 36 and submitted to GEO repository under super series GSE77252 gathering GSE90764 series for small RNA sequencing of undifferentiated and fully differentiated HAECs (*n* = 3 donors), GSE77249 series for small RNA sequencing of synthetic microRNA transfection, and GSE77251 for transcriptome analysis in HAECs overexpressing canonical miR‐34abc/449abc, 5′‐isomiR‐34b, or 5′‐isomiR‐449c.

### Data analysis

SOLiD data were analyzed with lifescope v2.5.1, using the small RNA pipeline for miRNA libraries and whole transcriptome pipeline for RNA‐seq libraries with default parameters. Annotation files used for production of raw count tables correspond to miRBase v18 for small RNAs and Refseq Gene model v20130707.

The abundance of isomiRs was quantified using custom java program. For each miRNA from miRBase v18, we extracted the number of reads starting at the exact miRBase position versus the number of reads starting at position −2, −1,+1, and +2 from the miRBase start position, then we computed for each miRNA the percentage of reads mapping to each isomiR.

Data generated from RNA sequencing were analyzed with Bioconductor (http://www.bioconductor.org). Differential expression analyses were performed using the edger package V 3.14.0 (Bioconductor). Briefly, dispersion was estimated using the parameters method = ‘deviance’ and robust = TRUE, and statistical significance was assessed using exact tests. *P*‐values were adjusted for multiple testing using the Benjamini and Hochberg method which controls the false discovery rate (FDR) 37. Thousand one hundred and ninety‐six genes down‐regulated in at least one condition compared to control were selected based on an average expression level log_2_ CPM (counts per million) > 3 and a log_2_ FC (fold change) ≤ −0.9 in at least one condition. Log_2_ CPM data were centered and scaled per gene. An unsupervised hierarchical clustering was performed with the R package pheatmap using Pearson's correlation distance and complete linkage.

The relative enrichment of sequences in the 3′‐UTR in gene lists from GSE77251 and ranked by increasing fold change was identified using sylamer 38 version 08‐123; online available at www.genomique.info/sylamer.

‘MicroCible’ is a bioinformatics tool developed in‐house to predict miRNA targets (http://www.genomique.info:8080/merge/index?action=MICIB, 39) by scanning transcripts sequences for the presence of ‘miRNA seed’ complementary sequence. This search can be performed for different ‘seed’ match type, a minimal free energy binding cutoff (deltaG≤ −20 was used for Fig. 2B,C), and the location of the potential targeting site (i.e. 3′UTR or entire transcript).

### Quantitative RT‐PCR

Quantitative RT‐PCR was performed using TaqMan^®^ Gene Expression Assay (Life Technologies) on a Lightcycler 480 (Roche, Basel, Switzerland) according to the manufacturer's instructions. Expression levels of messenger RNAs were calculated using the 2‐deltaCT method, using *UBC* as endogenous controls.

### Immunocytochemistry

A549 cells were grown on glass coverslips for detection of focal adhesion using anti‐paxillin antibody. Cells were then fixed (4% paraformaldehyde, 15 min, 4 °C), rinsed (PBS‐glycine 0.1 m, 10 min), and permeabilized (0.1% Triton X‐100, 5 min). Fixed cells were blocked for 1 h in 3% BSA and incubated for 1 h at room temperature with anti‐paxillin primary antibody (1 : 10 000, 349/Paxillin, BD Biosciences, San Jose, CA, USA). Then, cells were incubated for 1 h with the appropriate secondary antibody (Alexa Fluor^®^, 1 : 500, Invitrogen), nuclei were stained with DAPI (300 nm, Invitrogen), and F‐Actin was stained with Alexa Fluor^®^ 594 Phalloidin (1U/staining). Stained cells were mounted with ProLong^®^ Gold antifade reagent (Invitrogen, Life technologies). Images were acquired using the Olympus Fv10i confocal imaging systems (Olympus France, Rungis, France).

### Western blot experiments

Primary HAECs were harvested by scraping in Ripa lysis buffer (Thermo Scientific Pierce) and cleared by centrifugation. Protein concentration was determined using the bicinchoninic acid assay (Thermo Fisher Scientific) and equivalent amounts of protein were resolved on SDS polyacrylamide gels using Novex^®^ NuPAGE^®^ SDS/PAGE Gel System following the manufacturer's instructions. Proteins were transferred to PVDF membranes and analyzed by immunoblotting with appropriate primary antibodies and HRP‐conjugated secondary antibodies (1 : 500, Dako, Agilent Technologies) according to the manufacturer's instructions. Primary antibodies and dilution used were as follows: ARHGAP1 [1 : 500, Santa Cruz Biotechnology (Dallas, TX, USA) sc‐68918], ARHGDIB [1 : 500, Abcam (Cambridge, UK), Ab15198], R‐Ras (1 : 500, Santa Cruz, sc‐523), and Notch1 (1 : 500, Abcam, Ab27526). Immunoreactive bands were detected using immobilon ECL kit (Merck Millipore, Billerica, MA, USA) on a LAS‐3000 imager (Fujifilm France, BOIS D'ARCY, France).

### Plasmid constructs and luciferase measurements

Sequence from the wild‐type 3′‐UTR of *ARHGAP1, ARHGDIB*,* RRAS*, and *NOTCH1* was synthesized (gBlocks^®^ Gene Fragments, Integrated DNA Technologies, Coralville, IA, USA) and cloned into psiCheck2 vector (Promega, Madison, WI, USA). HEK293T cells were seeded on 96‐well plate at 2.10^4^ cell per well. The next day, psiCheck2 constructions (200 ng) were cotransfected with synthetic microRNA mimics (33 nm) (Ambion, Applied Biosystems, Foster City, CA, USA) into HEK293T cells, and luciferase activity was measured at 48 h post‐transfection using the dual reporter luciferase assay kit (Promega), according to the manufacturer's protocol.

### Cell cycle analysis

Fluorescence ubiquitin cell cycle indicator (FUCCI)‐expressing A549 cell lines were generated and used as previously described 40. FUCCI cells exploit the regulation of cell cycle‐dependent ubiquitination to label individual nuclei in G1 phase red, and those in S/G2/M phases green 41. Replication‐defective, self‐inactivating retroviral constructs were used to establish a stable A549 FUCCI‐2A cell line. The pPRIPu CrUCCI plasmid was obtained from C. Feillet and F. Delaunay. Nonmarked: Early G1, Kusabira‐Orange 2 (mKO2) only: G1, mKO2 + Azami‐Green 1 (mAG1): Early S, mAG1 only: S/G2/M. mKO2 and mAG1 were excited with 561 nm and 488 nm laser lines, respectively. Fluorescence was collected at 585 nm (585/15 band‐pass) for mKO2 and at 530 nm (530/30 band‐pass) for Geminin. FUCCI‐A549 cells were transfected 24 h after seeding with either miR‐Neg, canonical miR‐34/449, 5′‐isomiR‐34b, or 5′‐isomiR‐449c at 10 nm using Lipofectamine RNAi Max Reagent (Invitrogen) in OPTIMEM (Invitrogen) according to the manufacturer's instructions. Cellular fluorescence was then assessed 72 h after transfection by microscopy (LSM 780, Zeiss). Using imagej software, fluorescent cells were counted positive for one color (corresponding to cell cycle phases) if the fluorescence reached a defined threshold.

## Results and Discussion

### Identification of 5′‐isomiRs of miR‐34b and miR‐449c

**Figure 1:**
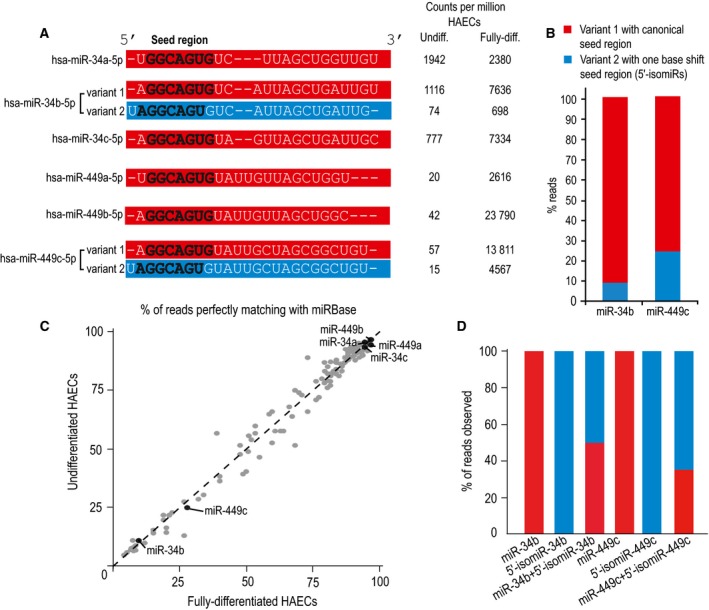
Identification of isomiRs of hsa‐miR‐34b‐5p and hsa‐miR‐449c‐5p. (A) Sequences from every mature variant of the miR‐34/449 family are illustrated. Bold letters correspond to the seed region (nucleotide 2–8). The sequences with the canonical seed region are in red. For hsa‐miR‐34b and hsa‐miR‐449c, the miRBase reference sequences with the one‐base shift seed region (variants 2) are in blue, whereas the variants 1 with the canonical seed region are in red. Small RNA sequencing experiments were performed from primary undifferentiated and fully differentiated HAECs. The counts per million (CPM) for each miR‐34/449 sequence obtained in each condition are indicated. Data are representative of three donors (GSE90764). (B) Histogram representing the percent of reads obtained by small RNA deep sequencing for both mature sequence variants of miR‐34b and miR‐449. (C) The graph displays the percentage of reads showing a perfect match with the reference miRNA sequences from miRBase, relative to the total number of reads. All miRNAs generating at least 30 counts in undifferentiated and fully differentiated HAEC small RNA libraries are shown (three replicates per condition, *R* ^2^ = 0.98). (D) 5′‐phosphate miRNAs, corresponding to the miRBase reference for hsa‐miR‐34b‐5p and hsa‐miR‐449c‐5p and their major variants were sequenced alone in a complex mixture of RNA (GSE77249). The histogram shows the percentage of reads observed for each unique miRNA sequence or for an approximate mix 1 : 1 between canonical miR and isomiR.

The miR‐34/449 family is highly expressed in airway multiciliated cells where it controls multiciliogenesis through regulations of several pathways 15, 17, 18, 19, 42. We examined the sequences of each miR‐34/449 member in small RNA sequencing experiments derived from undifferentiated and fully differentiated HAECs (Fig. 1A). Corresponding GEO references are provided in the Material and Methods section. We detected 5′ and 3′ variants in both 5p and 3p arms of miR‐34/449 sequences. As 3′ variants encompassed heterogeneous sequences and that mature miR‐34/449 of the 5p arm have been functionally established as evolutionary conserved multiciliogenesis regulators 15, 17, 18, in the present study we focused on 5′ variants of the 5p strand of miR‐34/449. Thus, we identified two distinct 5′ variants in miR‐34b and miR‐449c sequences (Fig. 1A–C). The most abundant variants account for 92% and 75% of the reads for miR‐34b and miR‐449c, respectively (Fig. 1A–C). These variants display a seed region that perfectly aligns with the rest of miR‐34/449 family (Fig. 1A). The least abundant variants account for 8% and 25% of the reads for miR‐34b and miR‐449c, respectively (Fig. 1A–C). They exhibit an extra uridine at their 5′‐end, which adds one base to their seed region compared to the rest of the family (Fig. 1A). We suspected that such a shift in the seed region could alter the target recognition and the biological properties of these two miRNAs. Intriguingly, miRBase defined the two sequences that contain an additional uridine in the 5′‐end as the mature forms of miR‐34b and miR‐449c. Based on these observations, we reassigned the least abundant forms as 5′‐isomiR variants. In the remaining of the present manuscript, we define them as 5′‐isomiR‐34b and 5′‐isomiR‐449c. At the same time, the best aligned miRNAs, previously considered as isomiRs by miRBase, should be considered as *bona fide* canonical miRNA sequences. In line with this statement, we first explored the sequence of these miRNAs in other species. This revealed that the 5′‐supplemental base was unique to human for miR‐449c. A different situation stands for miR‐34b, for which the 5′‐supplemental base was also reported in several other species including *Mus musculus*,* Xenopus tropicalis*,* Danio rerio*, and *Gallus gallus*.

We then reasoned that an incorrect definition of isomiRs observed for the miR‐34/449 family may also happen in other miRNA families. We thus reanalyzed small RNA sequencing experiments from 10 other human tissues, where we assessed the relative expression of miRNAs and isomiRs (Study SRP006043). By setting a detection threshold of 20 counts per million, we identified 80 miRNA sequences differing by an alternative 5′ shift form in at least 50% of their reads ( Table S1).

We excluded that this difference could come from a bias introduced during molecular cloning by designing and synthesizing four 5′‐phosphate miRNAs, corresponding to miR‐34b and 5′‐isomiR‐34b, miR‐449c, and 5′‐isomiR‐449c. Different proportions of these four miRNAs were exogenously added to a complex mixture of RNA, which was then sequenced. All sequences were appropriately identified in every tested condition (Fig. 1D). When the two different variant sequences of the same miRNA were mixed with a 1 : 1 ratio, they were roughly sequenced at the same proportion. We concluded from this test that our initial observations were not due to a preferential cloning.

A variable Drosha/Dicer cleavage has also been reported for some isomiRs 29, and this alteration depends on the different cell types and physiological conditions 21. We observed similar ratios between a large number of canonical miRNAs and isomiRs in independent samples, corresponding either to undifferentiated or fully differentiated HAECs (Fig. 1C). This suggests that the state of cellular differentiation has no detectable impact on the production of miR‐34/449 isomiRs. Incidentally, the lower abundance of isomiRs compared to canonical miRNA sequences is likely not the result of a weaker stability of variants sequences, as 5′‐isomiR‐34b and 5′‐isomiR‐449c sequences start with one uridine, usually associated with more stable sequences 43.

### IsomiR and canonical sequences of miR‐34b and miR‐449c exhibit distinct gene expression profiling

**Figure 2:**
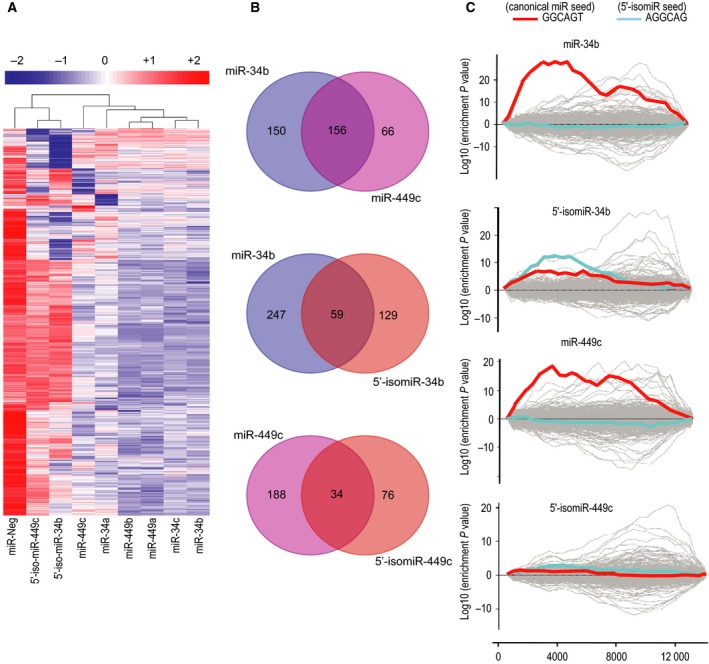
Overexpression of canonical miRNAs or isomiRs induces distinct gene expression patterns. (A) Heatmap of gene expression of the 1196 down‐regulated genes (log_2_ FC≤ −0.9 and log_2_ CPM>3) between proliferating HAECs overexpressing each of the eight canonical miRs or isomiRs of the miR‐34/449 family compared to a control miRNA (miR‐Neg). Pearson's correlation distance was used both for genes and samples, and pairwise average‐linkage was used as clustering method. (B) Predicted target genes either for canonical miR‐34b, miR‐449c, 5′‐isomiR‐34b, or 5′‐isomiR‐449c were selected (see Materials and Methods). Venn diagrams show the number of predicted gene targets for each canonical miRNA or isomiR that were down‐regulated (log_2_ FC≤ −0.9 and log_2_ CPM>3) following 48‐h overexpression of canonical miR‐34b (blue circle), miR‐449c (magenta circle) versus 5′‐isomiR‐34b (red circle) or 5′‐isomiR‐449c (red circle), as indicated upon each circle. The intersection of two circles indicates the number of predicted targets commonly down‐regulated in both conditions as indicated. (C) Individual miRNA mimics for miR‐34b, miR‐449c, 5′‐isomiR‐34b, or 5′‐isomiR‐449c were transfected (10 nm, 72 h) in proliferating HAECs to examine target recognition induced by either canonical miRs or isomiR variants using RNA sequencing data (GSE77251) analyzed with Sylamer 38. The graphs illustrate the enrichment landscape plots for the presence of seed complementary hexamers in the 3′‐UTRs of down‐ or up‐regulated transcripts following transfection of individual miR mimics as indicated. Canonical miR‐34b or miR‐449c sites are represented with red lines and 5′‐isomiR‐34b or 5′‐isomiR‐449c sites are represented with blue lines. Gray lines served as negative controls in addition to all random 7‐mers. The *x*‐axis represents the ranked gene lists ordered by increasing fold change.

The robust expression of miR‐34/449 in airway multiciliated epithelial cells (up to 15% of total miRNA counts) 15, 17, 19 suggests that their 5′‐isomiR variants, that account for up to 8% of total miR‐34/449 sequences (Fig. 1A), may still alter gene expression. As 5′‐isomiRs may target very different sets of transcripts, they can confer additional mRNA regulatory properties 44, 45, 46. We thus examined the impact of the one‐base shift in the seed region of 5′‐isomiR‐34b and 5′‐isomiR‐449c on gene expression. Primary HAECs were transfected with each miRNA member of the miR‐34/449 family, including 5′‐isomiR‐34b and 5′‐isomiR‐449c, and the resulting gene expression profiles were analyzed using RNA sequencing and *in silico* approaches. Figure 2A shows that 5′‐isomiR‐34b and 5′‐isomiR‐449c induced distinct effects on global transcriptome compared with their canonical counterparts and the rest of the canonical members of the family (miR‐34a, miR‐34c, miR‐449a, and miR‐449b). Canonical miR‐34b and miR‐449c were clustered together with the rest of the canonical members of the family. They shared a higher number of predicted targets that were repressed compared to their isomiR counterparts (Fig. 2A,B, Table S2). Among the 306 and 222 predicted target genes and down‐regulated by canonical miR‐34b and miR‐449c, respectively, 156 were inhibited by both miR‐34b and miR‐449c. Figure 2B also shows that canonical miR‐34b and miR‐449c which share the same seed region may regulate several different targets. This suggests that the target definition is not entirely based on the seed region, and may also involve sequence outside of the seed region. Among 306 and 188 predicted target genes down‐regulated by miR‐34b and 5′‐isomiR‐34b, respectively, 59 were inhibited by both miR‐34b and 5′‐isomiR‐34b. Among 222 and 110 predicted target genes down‐regulated by miR‐449c and 5′‐isomiR‐449c, respectively, 34 were inhibited by both miR‐449c and 5′‐isomiR‐449c (Fig. 2B). The number of predicted targets that were repressed after isomiR overexpression was reduced by almost half relative to their canonical counterparts. This result illustrates the impact of a one‐base shift in the seed region, and points out to the fact that isomiR variants and canonical miRNAs do not necessarily regulate the same targets.

As currently available target prediction tools probably underestimate the impact of isomiRs upon target gene regulation, we also used Sylamer 38 to computationally identify overrepresented sequences in the 3′‐UTRs of down‐regulated mRNAs. Figure 2C shows that canonical miR‐34b or miR‐449c overexpression resulted in detection of enrichments only for the canonical seed matches (2–7, ‘GGCAGU’), though they also induced a down‐regulation of some 5′‐isomiR‐34b predicted targets (Fig. 2B) that were not detected using the Sylamer algorithm. Conversely, 5′‐isomiR‐34b overexpression enriched detection for both isomiR seed matches (2–7, ‘AGGCAG’ corresponding to 3–8 for the canonical seed), and for the canonical seed matches (2–7, ‘GGCAGU’) but to a lesser extent. Unexpectedly, we failed to detect any enrichment for canonical or isomiR seed matches in response to 5′‐isomiR‐449c overexpression (Fig. 2C). The lower number of target transcripts down‐regulated in response to this isomiR precluded their detection using Sylamer. Altogether, these results indicate that 5′‐isomiRs do interact with their ‘AGGCAG’ seed region, and also to some extent with the canonical seed region ‘GGCAGU’.

### Mature miRNAs and isomiRs target distinct transcripts

**Figure 3:**
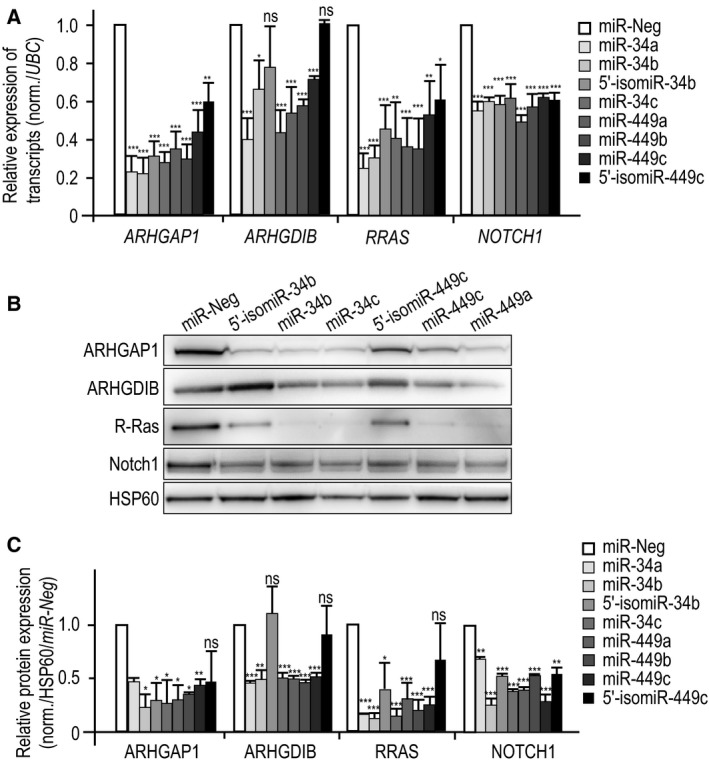
Specific targeting for canonical miRNAs and isomiRs. (A–C) Proliferating HAECs were transfected for 48 h with either canonical miR‐34/449, 5′‐isomiR‐34/449, or negative control miR‐Neg, as indicated on the figure. The effect of canonical miR‐34/449 or 5′‐isomiR‐34/449 overexpression on transcript (A) or protein (B, C) expression levels of ARHGAP1, ARHGDIB, Notch1, and R‐Ras, using real‐time RT‐PCR and western blot, were compared to miR‐Neg. Transcript expression levels were normalized using *ubiquitin C* gene (*UBC*). Blots are representative of 3–6 independent experiments. (C) The histogram illustrates the quantification of protein level of ARHGAP1, ARHGDIB, Notch1, and R‐Ras in response to canonical miR‐34/449, 5′‐isomiR‐34b, and 5′‐isomiR‐449c as normalized fold changes compared to miR‐Neg. Protein expression levels were normalized with an antibody against HSP60 as a loading control. Data are means ± SD from three to six donors (***, *P* < 0.001; **, *P* < 0.01, *, *P* < 0.05, ns, not significant, Student's *t*‐test).

**Figure 4:**
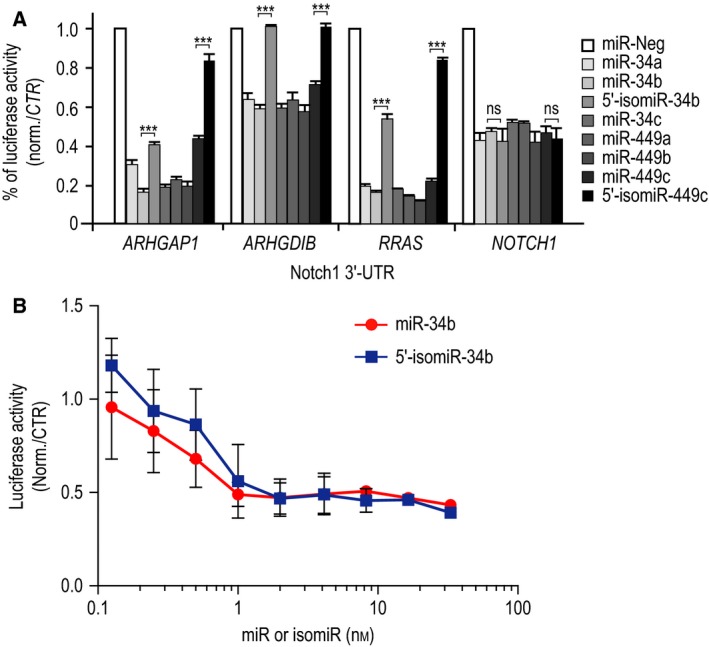
Specific interaction between either canonical miRNAs or isomiRs of the miR‐34/449 family and the 3′‐UTRs of validated targets. (A) Specific interaction between miR‐34/449 or 5′‐isomiR‐34/449 and the 3′‐UTRs of *ARHGAP1*,*ARHGDIB*,*RRAS*, and *NOTCH1* was confirmed using luciferase reporter assay on constructs carrying the wild‐type 3′‐UTR‐binding sites of canonical miR‐34/449 and isomiR‐34/449. Values were normalized to the miR‐Neg conditions. Data are means ± SD from five independent experiments. (B) Dose–response relationship of canonical miR‐34b or 5′‐isomiR‐34b was performed using luciferase reporter assay on the wild‐type 3′‐UTR of *NOTCH1*. Data are means ± SD from three independent experiments; ***, *P* < 0.001; **, *P* < 0.01, *, *P* < 0.05, ns, not significant, Student's *t*‐test.

Next, we explored the inhibitory effects of canonical miR‐34/449 and their isomiR variants on several miR‐34/449 targets, including ARHGAP1, ARHGDIB, R‐Ras, and Notch1, which we previously described to be involved in MCC differentiation 15, 19, 47. After examining their expression level in several RNA sequencing datasets (Fig 2), these targets were validated using quantitative RT‐PCR, western blot, and luciferase reporter assay in primary HAEC cultures, A549 cells, and/or HEK293 cells (Figs 3, 4). Figure 3 shows that canonical miR‐34b and miR‐449c induced similar effects on these miR‐34/449 targets compared to other canonical miR‐34/449 members. On the opposite, 5′‐isomiR‐34b and 5′‐isomiR‐449c, i.e. the sequences referenced in miRBase, exhibited much weaker effects on targets related to small GTPase pathway (ARHGAP1, ARHGDIB, and R‐Ras) (Figs 3, 4). 5′‐isomiRs had no significant effect on ARHGDIB, while a lesser robust inhibitory effect was noticed on ARHGAP1 and RRAS compared to their canonical counterparts. Both 5′‐isomiR‐34b and 5′‐isomiR‐449c induced similar inhibitory effects either on *RRAS* transcript level, RRAS protein level, or 3′‐UTR‐RRAS luciferase activity reporter assay (Figs 3, 4). Conversely, canonical miR‐34/449 induced twofold higher inhibitory effects on RRAS protein level and luciferase reporter assay than on *RRAS* transcript level. These results may suggest that canonical miR‐34/449 exert an inhibitory effect on RRAS protein expression without systematically inducing mRNA degradation. By analyzing the *RRAS* 3′‐UTR using the miRNA target prediction tool ‘microCible’, we observed that it contains three sites for canonical miR‐34/449 plus another site that is shared by both canonical miR‐34/449 and isomiR‐34/449. These results may suggest that the site shared by both canonical miR‐34/449 and 5′‐isomiR‐34/449 might be mainly involved in mRNA degradation, whereas the three other canonical sites might rather be involved in translation inhibition. We also observed a weaker effect of canonical miR‐449c compared to canonical miR‐34b on *RRAS* transcript level. In addition, as shown in Fig. 3A, we observed a significant difference between canonical miR‐449c and 5′‐isomiR‐449c on their inhibitory effects on *RRAS* transcript level. Conversely, no significant difference was observed between canonical miR‐34b and 5′‐isomiR‐34b on *RRAS* transcript level. These observations suggest again that some differences between sequences of canonical miR‐34b and miR‐449c outside the seed region (see Fig. 1A) may lead to some different properties of inhibition and targeting. The same observation may also apply for 5′‐isomiR‐34b and 5′‐isomiR‐449c.

On the contrary, 5′‐isomiR‐34b and 5′‐isomiR‐449c exerted similar inhibitory effects on the Notch pathway receptor Notch1 than their canonical counterparts (Figs 3, 4). A dose–response relationship using luciferase reporter assay also indicated that Notch1 was similarly repressed by either canonical miR‐34b or 5′‐isomiR‐34b at any tested concentrations (Fig. 4B). These results indicate that 5′‐isomiR‐34/449 were less efficient than miR‐34/449 to silence the expression of several well‐validated targets. Nevertheless, we found that Notch1, a key target that must be repressed by canonical miR‐34/449 to trigger multiciliogenesis 15, was one of the targets to be still repressed by isomiR ‐34/449. That is in line with models proposing that isomiRs may act cooperatively with their canonical miRNAs to target the same mRNAs or common signaling pathways to control the same biological functions 30.

Several studies already indicated a putative association between isomiRs and Argonautes (AGO) protein complexes 30, 48, 49. As overexpression of 5′‐isomiR‐34/449 induced significant effects on many targets including Notch1, isomiRs are clearly loaded in AGO protein complexes. The similar dose–response relationships observed for miRs and isomiRs on Notch1 repression suggest an equivalent loading into the RNA‐induced silencing complex (RISC).

### Canonical miRNAs and isomiR variants of miR‐34/449 family have distinct biological function

**Figure 5:**
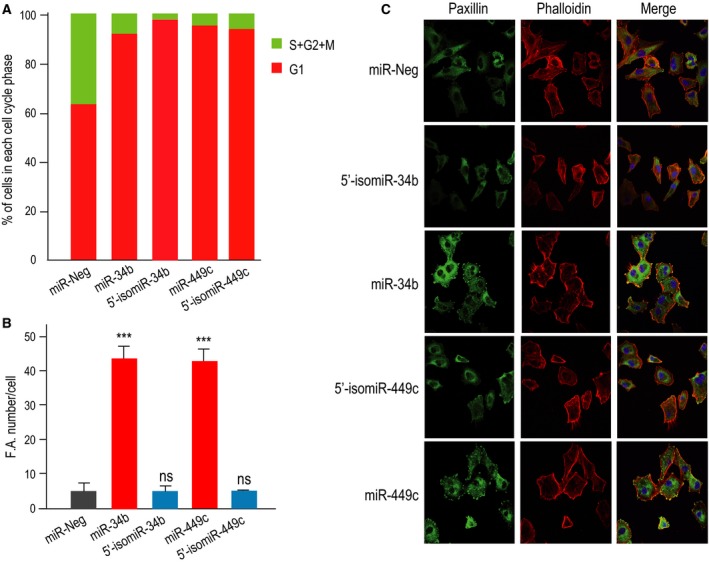
Functional differences between canonical miR‐34/449 and 5′‐isomiR‐34/449. (A) The bargraph illustrates the effects of expression of canonical miR‐34b or miR‐449c and 5′‐isomiR‐34b or 5′‐isomiR‐449c on the different phases of the cell cycle assessed using the FUCCI sensor cells. Red bars correspond to G1 phase and green bars correspond to S/G2/M phases. Data were compared to miR‐Neg conditions and are representative of three independent experiments. (B) The histogram represents the quantification of focal adhesion foci per cell in response to overexpression of miR‐Neg in gray as negative control, canonical miR‐34b, or miR‐449c in red and 5′‐isomiR‐34b or 5′‐isomiR‐449c in blue. Data are means ± SD from three to six independent experiments; ***, *P* < 0.001; **, *P* < 0.01, *, *P* < 0.05, ns, not significant, Student's *t*‐test. (C) Immunostaining of focal adhesion protein Paxillin (in green), F‐actin (in red), and nuclei (in blue) in A549 epithelial cells transfected for 72 h with either control miRNA (miR‐Neg), canonical miR‐34b, canonical miR‐449c, 5′‐isomiR‐34b, or 5′‐isomiR‐449c.

The precise determination of the roles played by isomiRs is hampered by the limited tools available for modulating specifically the levels of isomiRs at a one‐nucleotide accuracy and measuring their effects 29, together with the overlapping and redundant nature of most miRNAs 50. Importantly, we have previously shown that miR‐34/449 control multiciliogenesis by repressing cell cycle and Notch pathway 15. They also control actin network reorganization and focal adhesion formation in airway epithelial cells by repressing small GTPase components such as R‐Ras, ARHGAP1, or ARHGDIB 47. We therefore wondered whether the weaker abundance in 5′‐isomiRs relative to their canonical counterparts may contribute to the overall biological effects by affecting additional targets associated with a common pathway, such as the cell cycle or the Notch pathway. miR‐34/449 expression was increased by 1000 times during multiciliogenesis, representing up to 15% of the total of expressed miRNAs 15. Besides, 5′‐isomiR‐34b and 5′‐isomiR‐449c represent up to 8% of the total of miR‐34/449 sequences in fully differentiated HAECs (Fig. 1C). This huge level of expression implies that the small proportion of 5′‐isomiR‐34/449 present in the cells may already be sufficient to exert some biological functions. Hence, we examined whether 5′‐isomiRs may share similar biological effects with canonical miR‐34/449. However, the strong sequence homology between canonical and isomiRs of miR‐34/449 miRNAs impeded to experimentally test whether 5′‐isomiR‐34/449 may also functionally control multiciliogenesis by directly repressing Notch pathway. We therefore focused our attention on two other relevant pathways, namely cell cycle and focal adhesion formation. To address this issue, 5′‐isomiRs and canonical miR‐34/449 were overexpressed in proliferating FUCCI‐A549, a human lung epithelial cell line (see Materials and Methods), on which we measured the effects on cell cycle (Fig. 5A) and on focal adhesion formation (Fig. 5B,C). Canonical and isomiR sequences of miR‐34/449 strongly reduced the number of cycling cells (Fig. 5A), indicating that they exert similar effects on cell cycle arrest. A different situation was observed for the formation of focal adhesion, which was observed with all canonical sequences of miR‐34/449, as previously shown 19 (Fig. 5), but not with 5′‐isomiR‐34b and 5′‐isomiR‐449c. These results are consistent with previous observations indicating that the small GTPase components RRAS, ARHGDIB, and ARHGAP1, previously described to control actin network reorganization 19, were not or weakly repressed by isomiRs. The absence or decrease of inhibitory effects of isomiRs on small GTPase targets may thus explain the absence of functional effects of isomiRs on actin network. Altogether, our data indicated that isomiRs and canonical sequences of miR‐34/449 may share some biological effects but may also have distinct functions.

More generally, the overlapping targeting of targets and the high correlation between isomiRs and their canonical miRNAs may emphasize the impact of a given miRNA family on specific biological phenotypes. Among the small number of mRNAs predicted to be targeted and repressed by isomiRs, our data revealed key and evolutionary conserved genes such as Notch1 that must be repressed to trigger multiciliated cell differentiation 15, 51. In this context, 5′‐isomiR‐34/449 could act as additional mechanisms to strengthen miRNA‐induced silencing effect on important target genes.

In conclusion, our work reports an incorrect annotation in miRBase for several miRNA sequences, such as hsa‐miR‐449c‐5p and hsa‐miR‐34b‐5p, which actually correspond to isomiR variants with distinct functional effects. As most of commercial miR mimics currently used for functional studies are primarily based on information provided in miRBase, our study highlights the importance of careful examination of genuine mature canonical miRNA sequences. These differing sequences may profoundly affect the interpretation of many results currently available in the literature. Through the specific example of miR‐34/449 family, our study also suggests that isomiRs variants can share biological function and/or have distinct roles from their canonical homologs. Finally, our findings highlight how isomiRs may bring additional mechanisms by which miRNA machinery may exert a fine‐tune regulation of a complex biological function.

## Author's contributions

OM performed and analyzed experiments and contributed to draft manuscript. AP, AP and KL performed bioinformatics analyses which were managed by KL. RNA sequencing experiments were carried out by VM, NP, and L‐EZ. AC and BC contributed to qPCR, western blotting and luciferase assay experiments. PB and JZ generated FUCCI‐A549 cells. GP participated to cell cycle experiments. BM designed, planned, and analyzed experiments. BM and PB coordinated and supervised the project, and obtained funding. BM and PB analyzed and interpreted data and wrote the paper. All authors commented on the manuscript.

## Supporting information


**Table S1.** miRNA expression and sequence distribution in ten tissues (Study SRP006043).

**Table S2.** First sheet: Gene expression profiling obtained in RNA sequencing after 72‐h overexpression of individual miR mimics (negative control miR‐Neg, canonical miR‐34a/b/c or miR‐449a/b/c, 5′‐isomiR‐34b or 5′‐isomiR‐449c) in proliferating primary HAECs (GSE77251) and analyzed using the edger package V 3.14.0 (see Materials and Methods).
